# Kimura disease accompanied with Nephrotic syndrome in a 45-year-old male

**DOI:** 10.1186/s13000-015-0277-1

**Published:** 2015-04-28

**Authors:** Yu Gong, Jun-Ying Gu, Sony Labh, Yu-Ling Shi

**Affiliations:** Department of Dermatology, Shanghai Tenth People’s Hospital, Tongji University School of Medicine, 301, Middle Yanchang Rd, Shanghai, 200072 China; Department of Dermatology, Tongji University School of Medicine, Shanghai, China

**Keywords:** Kimura disease, Nephrotic syndrome, Angiolymphoid hyperplasia with eosinophilia, Prednisone

## Abstract

Kimura disease (KD) is an uncommon chronic inflammatory disorder of unknown etiology, occurs mainly in Asian young males, presenting as subcutaneous growing masses, with a predilection for head and neck, with or without satellite lymphadenopathy. Herein, we report a case of an atypical manifestation of KD accompanied with NS in a middle-aged man, though the patient was clinically misdiagnosed previously. The diagnosis of KD can be difficult and misleading, so we must explore the main points of KD so as to prevent misdiagnosis.

## Letter to the editor

KD is an uncommon chronic inflammatory disorder of unknown cause, involving subcutaneous tissue, predominantly in the head and neck region. It can be associated with lymphadenopathy (both local and distant), obvious peripheral blood eosinophilia, and an elevated IgE level [1]. Most cases reported to date involve young Asian males, with most patients being aged between 20 and 40 years [2]. The incidence of KD with coexisting renal disease ranges from 10% to 60% [3,4]. Herein, we report an atypical manifestation of KD accompanied with nephrotic syndrome (NS) occurring in a 45-year-old man.

A 45-year-old Chinese man came to our department with a soft mass in medial aspect of his right upper arm. According to the patient, the mass appeared in 2003 for the first time and that was without any obvious cause. He became aware of the mass after about 5 years when he noticed that the enlargement was gradually progressive. Then he visited general surgery department in October 2008 and underwent surgical resection of mass after 3 months in January 2009.

The histopathology report of the mass at that time revealed a proliferation of lymphoid follicles and diffuse infiltration of eosinophil, and WBC: 8200/dL (eosinophil: 38%). So it was diagnosed provisionally as a suspected case of parasitic infection by a physician, and then the patient wasn’t followed up.

**Figure 1:**
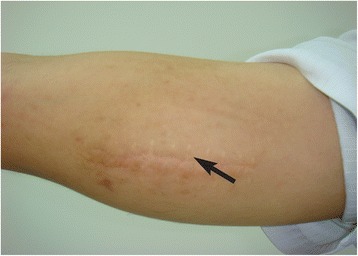
Clinical appearance of the case: a nontender firm subcutaneous swelling of 4 cm × 5 cm in size in the right upper arm region (arrow).

In 2013, the lesion reappeared and gradually became larger than before, so he visited our clinic. Physical examination revealed a firm, nontender, mobile, subcutaneous mass of 4 cm × 5 cm (Figure 1). The overlying skin was normal except for a 5 cm long scar (Figure 1). The patient’s blood pressure was 135/90 mmHg. The laboratory values were as follows: creatinine (2.4 mg/dL), normal electrolytes, low serum albumin (28 g/L), hypertriglyceridemia (3.2 mmol/L), proteinuria (3.5 g/d), WBC (8360/dL), eosinophilia (42% ), haemoglobin (13.6 g/dL), IgE (17100 IU/ml). Antinuclear antibody and other disease specific autoantibodies were negative. The bilateral lymph nodes (LN) of neck and axillae were enlarged. Investigations for a haematological malignancy including immunophenotyping of circulating lymphocytes, search for clonal T cell population and FIP1L1-PDGFRA fusion gene mutation, and examination of a bone marrow biopsy all gave negative results.

**Figure 2:**
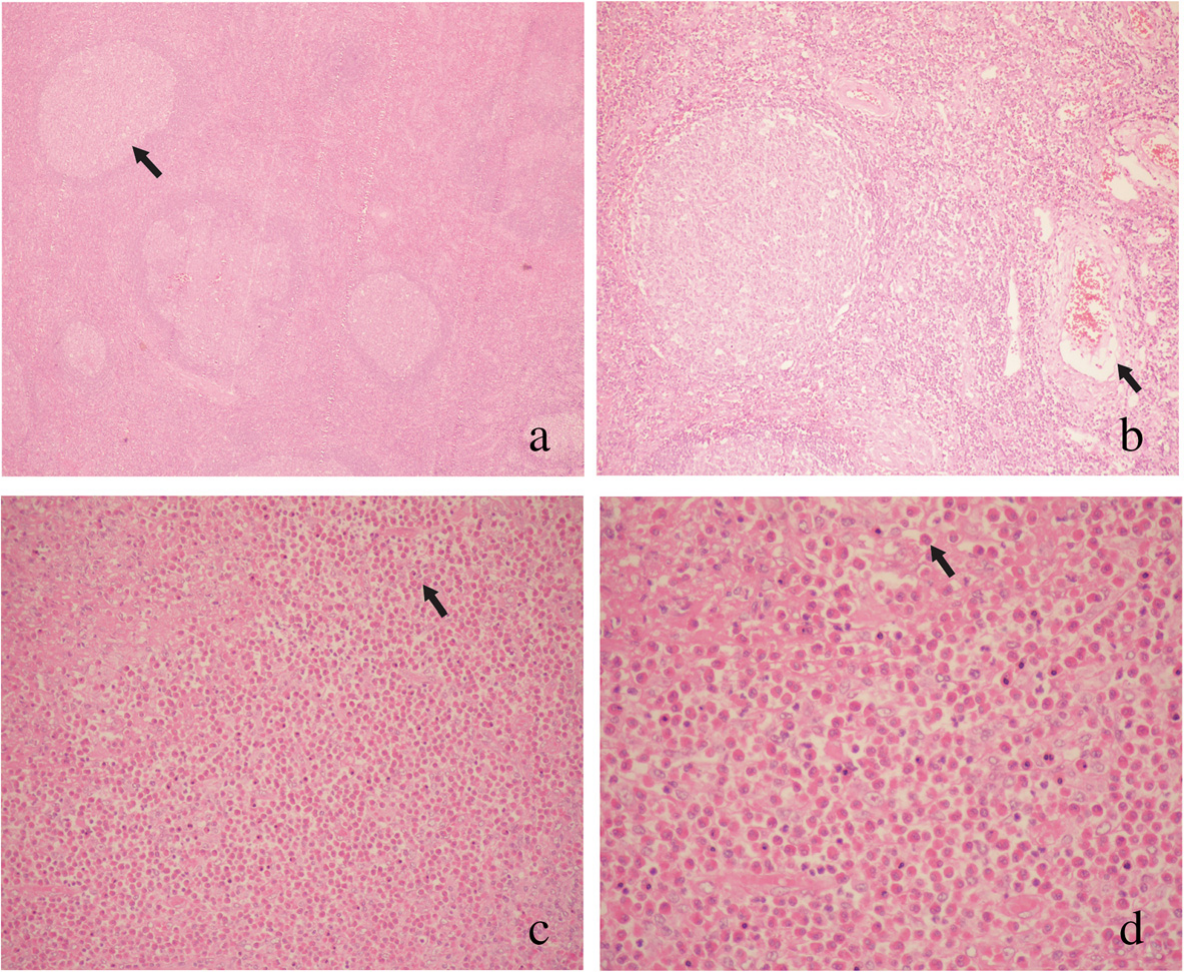
Histologic examination of a biopsy sample from the subcutaneous mass of the upper arm (H and E): **(a)** proliferation of lymphoid follicles with distinct germinal centres (arrow; × 40); **(b)** vascular proliferation (arrow; × 100); **(c, d)** formation of eosinophilic microabscess and intense eosinophilic infiltration (arrow; c × 200; d × 400).

Histological examination of a biopsy sample showed a proliferation of lymphoid follicles with distinct germinal centres (Figure 2a), capillary proliferation (Figure 2b), and diffuse infiltration of eosinophil and eosinophilic microabscess (Figure 2c and d).

The final diagnosis was KD accompanied with NS. But the patient refused the renal biopsy. After integrity surgical excision of the mass, he was treated with oral prednisone 30 mg per day for 4 months and responded well with normalization of eosinophil counts and IgE values, disappearance of proteinuria after 3 months. In addition, the enlarged LN shrunk to normal after 4 months. Currently, the patient is only on a low dose of oral prednisone (7.5 mg/d). There has been no recurrence of the disease for 1 year.

Association of KD with renal disease in the form of proteinuria and NS is well recognized. Proteinuria may occur in 12–16% of patients and 59–78% of them have a NS [3,4]. The renal pathologies reported in China have included minimal change disease, mesangioproliferative and membranous nephropathy, while a wider spectrum of histological lesions have been reported in other countries [5]. In our case, the patient refused the renal biopsy, so we didn’t know renal pathology.

KD is often confused with malignancies, T cell lymphoma, Hodgkin’s disease, parasitic infection and histiocytosis X. Ultrasound, CT, and magnetic resonance imaging (MRI) might be diagnostic and can help staging the extent and progression of the disease as well as the lymph node involvement. The peripheral blood eosinophilia can also mimic a parasitic infection or an allergic reaction. However, the lesion that bears the closest resemblance to KD is Angiolymphoid Hyperplasia with Eosinophilia (ALHE). The clinical and histological features of KD and ALHE are compared in Table 1 [6,7].

**Table 1:** **A comparison of the clinical and histological features of KD with ALHE**

	**Kimura disease**	**Angiolymphoid hyperplasia with eosinophilia**
**clinical features**		
Sex	Female predominance (70%)	Male predominance (85%)
Age	Young adulthood	Young to middle age
Race	More common in Asians	Occurs in all races
Location	Head and neck	Head and neck
Presentation	Localized subcutaneous mass	Dermal papules or nodules
Number	Single or multiple	Usually multiple
Size	Average 3 cm	Average 1 cm
Lymph node involvement	Common	Rare
peripheral eosinophilia	Almost invariably present	Rare (20%)
Serum immunoglobulin E (IgE) level	Elevated	Normal
Renal involvement	Occasional (21%)	Rare
Recurrence rate	30%	15-40%
**Histopathological features**		
Depth	Subcutaneous, muscle	Cutaneous, subcutaneous
Vascular proliferation	Some degree of vascular proliferation	Florid vascular proliferation
Lymphoid follicles	Always found	May be present
Eosinophils	Abundant	Sparse to abundant
Eosinophils abscesses	Present	Not seen
Endothelium	Flattened	Cuboidal to dome shaped:"Histiocytoid"
Fibrosis	Present	Absent

Treatment for KD includes surgical resection and regional or systemic steroid therapy [8]. Cytotoxic therapy and irradiation have also been utilized. Surgical excision of the lesion(s) is the first line therapy but relapses are frequent [5]. Systemic corticotherapy with prednisone is prescribed when renal involvement is present, but with a risk of relapse on withdrawal of medicine. In our case, our patient who is a middle-aged man presented with atypical KD with renal involvement that responded well to prednisone without relapse. The treatment resulted in rapid remission of NS along with the normalization of eosinophil count and IgE levels. The dose of prednisone had been tapered to 7.5 mg/d for 7 months and disease had not relapsed. He is still being followed-up.

## Consent

Written informed consent was obtained from the patient for the publication of this report and any accompanying images.
